# Protective Effects of SKF-96365, a Non-Specific Inhibitor of SOCE, against MPP^+^-Induced Cytotoxicity in PC12 Cells: Potential Role of Homer1

**DOI:** 10.1371/journal.pone.0055601

**Published:** 2013-01-31

**Authors:** Tao Chen, Jie Zhu, Chi Zhang, Kai Huo, Zhou Fei, Xiao-fan Jiang

**Affiliations:** 1 Department of Neurosurgery, Xijing Institute of Clinical Neuroscience, Xijing Hospital, Fourth Military Medical University, Xi'an, Shaanxi, China; 2 Department of Neurosurgery, The 123th Hospital of PLA, Bengbu, Anhui, China; Hertie Institute for Clinical Brain Research and German Center for Neurodegenerative Diseases, Germany

## Abstract

Parkinson's disease (PD) is the most common neurodegenerative movement disorder, characterized by loss of dopominergic (DA) neurons in substantia nigra pars compacta (SNpc), and can be experimentally mimicked by the neurotoxin MPP^+^ in vitro models. In this study, we investigated the potential protective effect of SKF-96365, a non-specific inhibitor of SOCE (store-operated calcium entry), on MPP^+^ induced cytotoxicity in PC12 cells. We found that pretreatment with SKF-96365 (10 µM and 50 µM) 30 min before injury significantly increased cell viability, decreased LDH release, prevented nuclear damage, and inhibited apoptotic cell death in MPP^+^ stressed PC12 cells. The results of calcium image using the ratiometric calcium indicator Fura-2-AM also showed that SKF-96365 reduced the intracellular calcium overload induced by MPP^+^ in PC12 cells. In addition, SKF-96365 decreased the expression of Homer1, a more recently discovered postsynaptic scaffolding protein with calcium modulating function, following MPP^+^ administration in PC12 cells, while had no statistically significant effects on endoplasmic reticulum (ER) calcium concentration. Furthermore, overexpression of Homer1 by using recombinant lentivirus partly reversed protective effects of SKF-96365 against MPP^+^ injury. The ER Ca^2+^ release was further amplified and ER calcium recovery was delayed by Homer1 upregulation in PC12 cells following MPP^+^ insult. Taken together, these data suggest that SKF-96365 protects PC12 cells against MPP^+^ induced cytotoxicity, and this protection may be at least in part on the inhibition of intracellular calcium overload and suppression of Homer1-mediated ER Ca^2+^ release.

## Introduction

Parkinson's disease (PD), the second most common chronic neurodegenerative disorder after Alzheimer's disease, is characterized primarily by the loss of dopaminergic (DA) neurons in the substantia nigra pars compacta (SNpc) and the formation of cytoplasmic “Lewy body” inclusions in some of the surviving neurons [1], [2]. With the progressive degeneration of DA neurons in SN and degeneration of the nerve terminals in the striatum, the concentration of striatal dopamine reduces to less than 70–80% of the normal level, which will eventually lead to the movement disorders, including bradykinesia, rigidity, resting tremor and postural instability [3]. Because of the ignorance as to the precise aetiology of PD, we still do not have clinical therapeutic measures to prevent nigral neuronal degeneration and disease progression for patients [4]. However, increasing evidence has demonstrated that abnormal calcium homeostasis is one of the fundamental processes contributing to DA neuronal death, and inhibition of calcium dysfunction through pharmacological or genetic methods could be a critical strategy for PD [5]–[7].

Cytosolic calcium acts as a ubiquitous second messenger and Ca^2+^-mediated cellular stress has long been thought to be important in neurodegenerative disease including PD [8]. The unusual reliance of DA neurons on calcium in autonomous pacemaking suggests that stress of storage organelles (such as mitochondria and endoplasmic reticulum) created by calcium homeostasis dysfunction could be responsible for their selective vulnerability, rather than simply a late-stage consequence [9], [10]. Calcium signals are generated by both the release of stored calcium from the endoplasmic reticulum and the influx of extracellular calcium across the plasma membrane. The entry of extracellular calcium generally results from depletion of intracellular stores in a process referred to as capacitative calcium entry or store-operated calcium entry (SOCE), which not only ensures optimal refilling of the endoplasmic reticulum, but also leads to a prolonged increase in cytosolic Ca^2+^ [11], [12].

The more recently discovered postsynaptic scaffolding proteins Homer, which express in various tissues but appear to be enriched in the CNS, play a central role in Ca^2+^ signaling [13]. They share a conserved N-terminal Ena/VASP homology 1 (EVH1) domain which functions as a protein–protein interaction motif to bind a proline-rich consensus sequence (PPXXFR) in various other scaffolding and signal transduction molecules, including metabotropic glutamate receptors (mGluRs), IP_3_ receptors (IP_3_Rs), Shank proteins, and TRPC channels [14], [15]. Although the role of TRPC channel-mediated calcium homeostasis in neuronal cell death is not fully understood, a previous study has shown that Homer1 protein (also known as Ves1) is required for gating of TRPC1 and regulates the communication between membrane TRPC channels and intracellular calcium stores, which are key factors of neuronal function [16]. The present study was designed to test the hypothesis that the non-specific SOCE inhibitor SKF-96365 would protect PC12 cells in an in vitro model of PD created by MPP^+^ insult. We found that SKF-96365 reduced MPP^+^-induced cell death and attenuated intracellular calcium overload in PC12 cells. Moreover, SKF-96365 significantly decreased the expression of Homer1 protein, and this protection might be associated with Homer1-dependent regulation of ER calcium homeostasis.

## Materials and Methods

### Cell culture and drug treatment

PC12 (adrenal gland; pheochromocytoma) cells were obtained from the Institute of Biochemistry and Cell Biology, SIBS, CAS [17]. The cells were maintained in poly-d lysine coated dishes in Dulbecco's modified Eagle's medium (DMEM) supplemented with 10% fetal bovine serum (Gibco, MD) and 1% antibiotics (penicillin/streptomycin) in a humidified 5% CO_2_ incubator at 37°C. SKF-96365 (sigma) was dissolved in 0.9% sterile saline water and added into the culture medium to achieve the final concentration of 1 µM, 10 µM or 50 µM 30 min before MPP^+^ treatment. DHPG (sigma) was dissolved in 0.9% sterile saline water to achieve the final concentration of 50 µM. The 0.9% sterile saline water was used in the Vehicle group cells.

### Cell viability assay

Cell viability assay was performed by using The Cell Proliferation Reagent WST-1 (Roche) following the manufacture's protocol. Briefly, PC12 cells were cultured in 96-well plates at a concentration of 0.5–5×10^4^ in a final volume of 100 µl/well culture medium. After various treatment, 10 µl cell proliferation reagent WST-1 was added into each well and incubated for 4 hours at 37°C and 5% CO_2_. Then, plates were shook thoroughly for 1 minute on a shaker and the absorbance of the samples were measured using a microplate ELISA reader. The absorbance of 100 µl culture medium plus 10 µl WST-1 mixed in one well in the absence of cells was used as a blank position for the ELISA reader. Cell viability was expressed as a percentage of the values of cells without any treatment.

### Lactate dehydrogenase (LDH) assay

Cytotoxicity was determined by the release of LDH, a cytoplasmic enzyme released from cells, and a marker of membrane integrity. LDH release into the culture medium was detected using a diagnostic kit (Jiancheng, nanjing) according to the manufacturer's instructions. Briefly, 50 µL of supernatant from each well was collected to assay LDH release. The samples were incubated with reduced form of nicotinamide-adenine dinucleotid (NADH) and pyruvate for 15 min at 37°C and the reaction was stopped by adding 0.4 mol/L NaOH. The activity of LDH was calculated from the absorbance at 440 nm and background absorbance from culture medium that was not used for any cell cultures was subtracted from all absorbance measurements. The results were normalized to the maximal LDH release, which was determined by treating control wells for 60 min with 1% Triton X-100 to lyse all cells.

### Hoechst and propidium iodide (PI) staining

For immuno-cytochemical staining, PC12 cells were seeded on PLL-coated glass slides at a density of 3×10^5^ cells/cm^2^. After various treatments, cells were fixed in 4% paraformaldehyde and stained with 10 µg/ml Hoechst 33342 or 10 µg/ml PI at 37°C for 10 min. Cells were then washed three times with PBS and the fluorescence was observed by using an Olympus BX60 microscope with the appropriate fluorescence filters. The number of Hoechst 33342-positive and PI-positive cells were counted in five fields in each section, and the results were expressed as the percentage of the values of MPP^+^-treated cells (Vehicle group).

### Flow cytometry

PC12 cells were harvested 24 h after exposure to MPP^+^, washed with ice-cold Ca^2+^ free PBS, and re-suspended in binding buffer. Cell suspension was transferred into a tube and double-stained for 15 min with Alexa Fluor 488-conjugated annexin V (AV) and PI at room temperature in the dark. After addition of 400 µl binding buffer, the stained cells were analyzed by an FC500 flow cytometer with the fluorescence emission at 530 nm and >575 nm. The CXP cell quest software (Beckman-Coulter, USA) was used to count the number of cells in B1 (AV^−^/PI^+^, the necrotic cells), B2 (AV^+^/PI^+^, the late phase apoptotic cells), B3 (AV^−^/PI^−^, normal cells) and B4 (AV^+^/PI^−^, the early phase apoptotic cells), and analyzed the results.

### Real-time RT-PCR

Total RNA was isolated 10 min after MPP^+^ insult using Trizol according to the manufacturer's instructions. A 2–3 µg template RNA was used to synthesize the first strand of cDNA using a reverse transcription kit purchased from Takara. Real-time PCR of cDNA was performed using the forward and reverse primer sequences: Homer1: forward, 5′-ACCTATCTTCAGCACTCGAGC-3′; reverse, 5′-CGTTGATACTTT -CCGGTGTTA-3′; GAPDH: forward, 5′-GGGTCAGAAGGATTCCTATG-3′; reverse, 5′-GGTCTCAAACATGATCTGGG-3′. Data were analyzed using a comparative critical threshold (Ct) method where the amount of target normalized to the amount of endogenous control and relative to the control samples.

### Lentivirus construction and transfection

The coding sequence of Homer 1 was amplified by RT-PCR. The primer sequences were: forward, 5′-ACCTATCTTCAGCACTCGAGC-3′; reverse, 5′-CGTTGATACTTTCCGGT -GTTA-3′. The PCR fragments and the pGC-FU plasmid (Shanghai GeneChem) were digested with Age I and then ligated with T4 DNA ligase to produce pGC-FU-Homer1. To generate the recombinant lentivirus LV-Homer1, 293T cells were co-transfected with of the pGC-FU plasmid (Shanghai GeneChem) (20 µg) with a cDNA encoding Homer 1, pHelper1.0 plasmid (15 µg) and pHelper2.0 plasmid (10 µg) by using Lipofectamine 2000 (Invitrogen) (100 µl). After 48 hours, supernatant was harvested from and the viral titer was calculated by transducing 293T cells. As a control, we also generated a control lentiviral vector that expresses GFP alone (LV-Con). PC12 cells were transfected with lentivirus vectors for 72 hours and subjected to various treatments.

### Calcium imaging

Intracellular Ca^2+^ concentration ([Ca^2+^]_cyt_) and Ca^2+^ in ER ([Ca^2+^]_ER_) were measured using the calcium indicator Fura-2-AM as previously described [18]. Cultured PC12 cells grown on glass slides were loaded with 5 µM fura-2 AM for 45 min before MPP^+^ treatment at room temperature. Cells were then placed in the open-bath imaging chamber containing Dulbecco's PBS (0.901 mM CaCl_2_, 0.493 mM MgCl_2_–6H_2_O, 2.67 mM KCl, 1.47 mM KH_2_PO_4_, 137.93 mM NaCl, and 8.06 mM Na_2_HPO_4_–7H_2_O, pH = 7.2–7.4) supplemented with 20 mM glucose at ambient temperature. Using the Nikon inverted epifluorescence microscope, cells were excited at 345 and 385 nm and the emission fluorescence at 510 nm was recorded. To determine [Ca2^+^]_ER_, the plasma membrane was permeablized with 30 s exposure to saponin (3.0 µg/mL) to eliminate the cytosolic fura-2 signal. This treatment caused a decrease in cytosolic mag-fura-2 fluorescence but an increase in the ratio of 345 nm/385 nm (F_345/385_, R_base_), which reflects fura-2 in ER. R_min_ was obtained with a minimum F_345/385_ ratio in a Ca^2+^-free solution (in mM, 25 NaCl, 125 KCl, 10 HEPES, 4 EGTA, 0.005 4-bromo A-23187). R_max_ was the maximum F_345/385_ ratio in a high Ca^2+^ solution (containing 10 mM CaCl_2_). The Ca2+ER values were then calculated using the equation [Ca^2+^_ER_] = *K*×(*R*_base_−*R*_min_)/(*R*_max_−*R*_base_). K was determined in neurons using solutions of known Ca^2+^ concentrations (Calcium Calibration Buffer Kit, Invitrogen, Carlsbad, CA, USA). Images were collected and analyzed with the Meta Fluor image-processing software. The [Ca^2+^]_cyt_ and [Ca^2+^]_ER_ values were then calculated and the Ca^2+^-insensitive fluorescence was subtracted from each wavelength before calculations.

### Western blot

PC12 cells in 6 cm dishes were washed with ice-cold PBS for three times and lysed with a lysis buffer containing protease inhibitors at 20 min after MPP^+^ insult. The protein concentration was determined using a BCA protein assay kit. Equivalent amounts of protein (40 µg per lane) were loaded and separated by 10% SDS-PAGE gels, and transferred to polyvinylidene difluoride (PVDF) membranes. Membranes were blocked with 5% nonfat milk solution in tris-buffered saline with 0.1% Triton X-100 (TBST) for 1 h, and then incubated overnight at 4°C with the primary Homer1 antibody (1∶1000) or β-actin antibody (1∶800) dilutions in TBST. After that the membranes were washed and incubated with secondary antibody for 1 h at room temperature. An analysis software named Image J was used to quantify the optical density of each band.

### Statistical analysis

Statistical analysis was performed using SPSS 16.0, a statistical software package. Statistical evaluation of the data was performed by one-way analysis of variance (ANOVA) followed by Bonferroni's multiple comparisons or unpaired *t* test (two groups). A value of *p*<0.05 was considered statistically significant.

## Results

### Effect of SKF-96365 on MPP^+^-induced cytotoxicity

**Figure 1:**
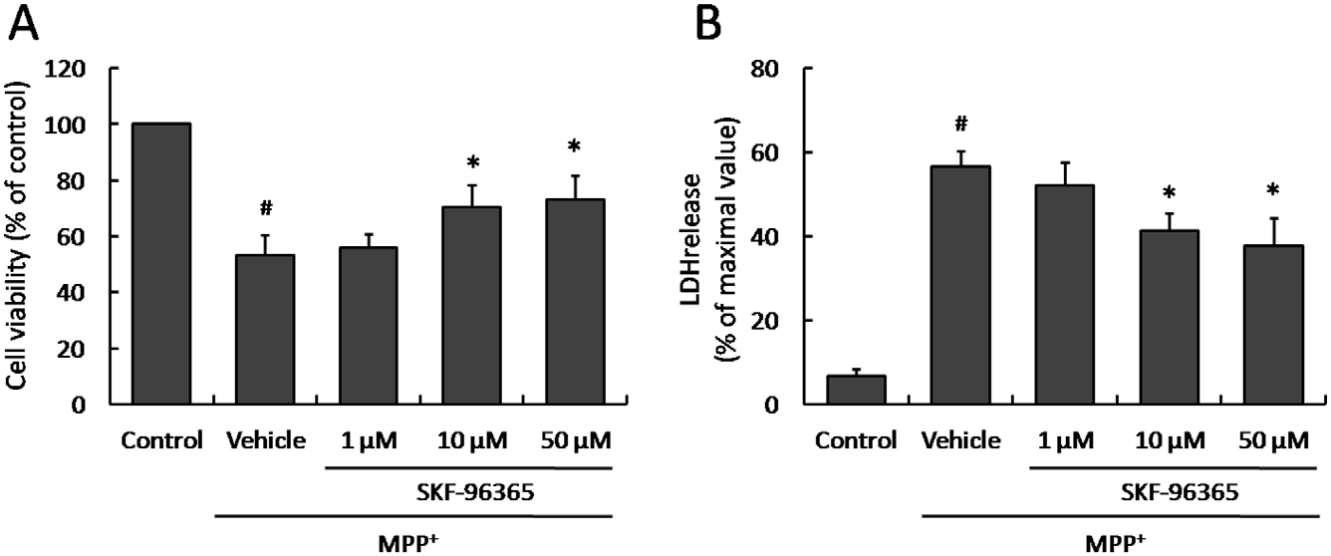
Effect of SKF-96365 on MPP^+^-induced cytoxicity. PC12 cells were pretreated with SKF-96365 in different concentrations (1 µM, 10 µM or 50 µM) 30 min before MPP^+^ insult, and the cell viability (A) and LDH release (B) were assayed 24 h later. The data were represented as means ± SD from five experiments. ^#^ *p*<0.05 vs. control group and ^*^ *p*<0.05 vs. Vehicle group.

To investigate whether SKF-96365 could protect PC12 cells from injury induced by MPP^+^ insult, cultured PC12 cells were pretreated with SKF-96365 in different concentrations (1 µM, 10 µM or 50 µM) 30 min before MPP^+^ addition. The cells viability was measured 24 h after MPP^+^ insult by using the cell proliferation reagent WST-1. It was found that SKF-96365 at the concentrations of 10 µM and 50 µM significantly inhibited the decrease of cell viability induced by MPP^+^ insult, although 1 µM SKF-96365 was not effective compared with Vehicle group (Fig. 1A). We also determined the release of LDH, a cytoplasmic enzyme released from cells and a marker of membrane integrity, in MPP^+^-insulted PC12 cells. We observed a similar protective effect on LDH release that SKF-96365 reduced the LDH release at the concentrations of 10 µM and 50 µM, not 1 µM (Fig. 1B). Because SKF-96365 in all concentrations mentioned before did not affect the cell viability and LDH release in normal PC12 cells (data not shown), 50 µM SKF-96365 was used in the following experiments.

### Effect of SKF-96365 on MPP^+^-induced cell death

**Figure 2:**
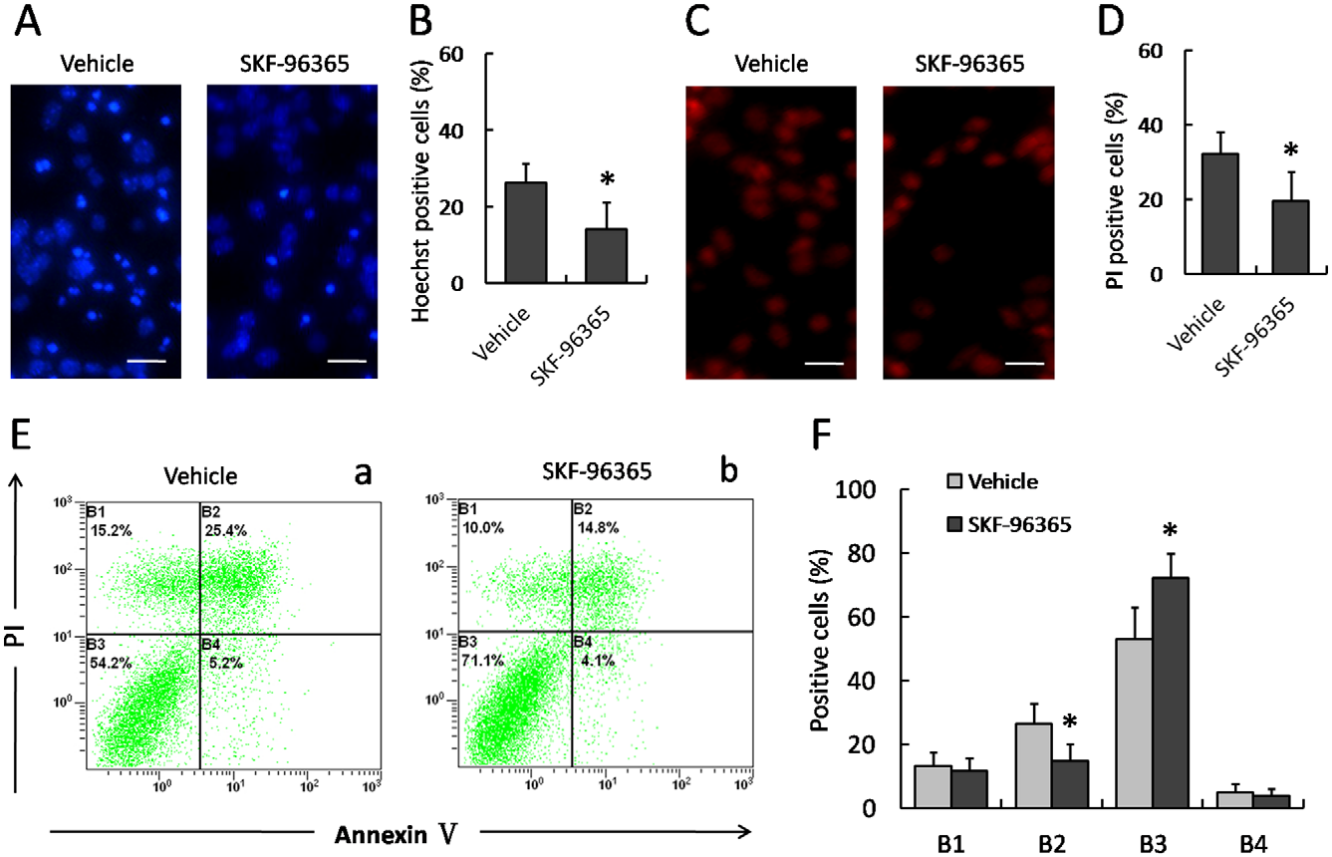
Effect of SKF-96365 on MPP^+^-induced cell death. PC12 cells were pretreated with 50 µM SKF-96365 or saline water (Vehicle) 30 min before MPP^+^ insult, and stained with Hoechst 33342 (A) or PI (C) 24 h later. The number of Hoechst-positive (B) and PI-positive cells (D) were calculated, respectively. The number of cells in B1 (AV^−^/PI^+^, the necrotic cells), B2 (AV^+^/PI^+^, the late phase apoptotic cells), B3 (AV^−^/PI^−^, normal cells) and B4 (AV^+^/PI^−^, the early phase apoptotic cells) were also analyzed using Flow cytometry (E and F). The data were represented as means ± SD from five experiments. ^*^ *p*<0.05 vs. Vehicle group.

Hoechst 33342 staining was performed to determined MPP^+^-induced nuclear damage of PC12 cells (Fig. 2A and 2B). In the Vehicle group, MPP^+^ insult caused DNA fragmentation and condensation of nuclear chromatin, while 50 µM SKF-96365 prevented these morphological changes in injured PC12 cells, indicating a protective effect of SKF-96365 on nuclear damage. We also detected membrane damage of PC12 cells by PI staining, and 50 µM SKF-96365 markedly decreased the number of PI-positive cells as compared to Vehicle group (Fig. 2C and 2D). In addition, flow cytometry was also examined to determine MPP^+^-induced apoptosis of PC12 cells (Fig. 2E). As shown in Fig. 2F, 50 µM SKF-96365 not only increased the number of AV^−^/PI^−^ cells, but also decreased the number of AV^+^/PI^+^ cells, suggesting an anti-apoptotic activity of SKF-96365.

### Effect of SKF-96365 on MPP^+^-induced intracellular calcium overload

**Figure 3:**
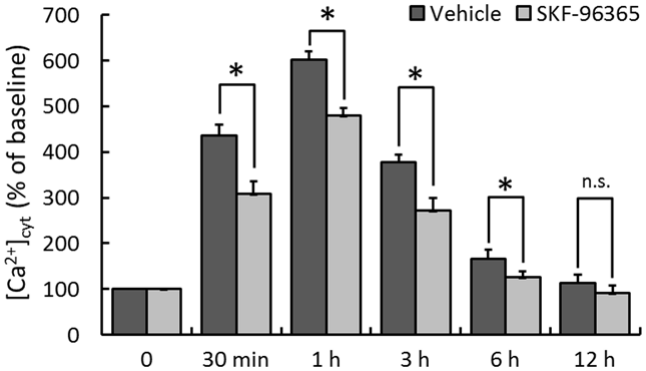
Effect of SKF-96365 on Ca^2+^ homeostasis in MPP^+^-treated PC12 cells. PC12 cells were pretreated with 50 µM SKF-96365 or saline water (Vehicle) 30 min before MPP^+^ insult, and the intracellular Ca^2+^ concentration ([Ca^2+^]_cyt_) was measured up to 12 h. The data were represented as means ± SD from eight experiments. ^*^ *p*<0.05 and n.s., not statistically significant.

To assess the potential role of intracellular Ca^2+^ homeostasis in SKF-96365 -induced protective effects, we monitored [Ca^2+^]_cyt_ using the ratiometric calcium indicator Fura-2-AM. Fig. 3 shows dynamic changes of [Ca^2+^]_cyt_, expressed as a percentage of the baseline for up to 12 h following MPP^+^ injury. MPP^+^ insult triggered a rapid rise in [Ca^2+^]_cyt_ within 1 h that slowly returned to the baseline in 12 h. Pretreatment with KSF-96365 significantly lower [Ca^2+^]_cyt_ at 30 min (437±23% vs 308±27%), 1 h (602±19% vs 479±17%), 3 h (378±17% vs 272±27%) and 6 h (166±19% vs 126±14%), as compared to Vehicle cells, indicating an delayed calcium afflux and an decreased Ca^2+^ overload after MPP^+^ insult.

### Involvement of Homer1 in SKF-96365-induced neuroprotection

**Figure 4:**
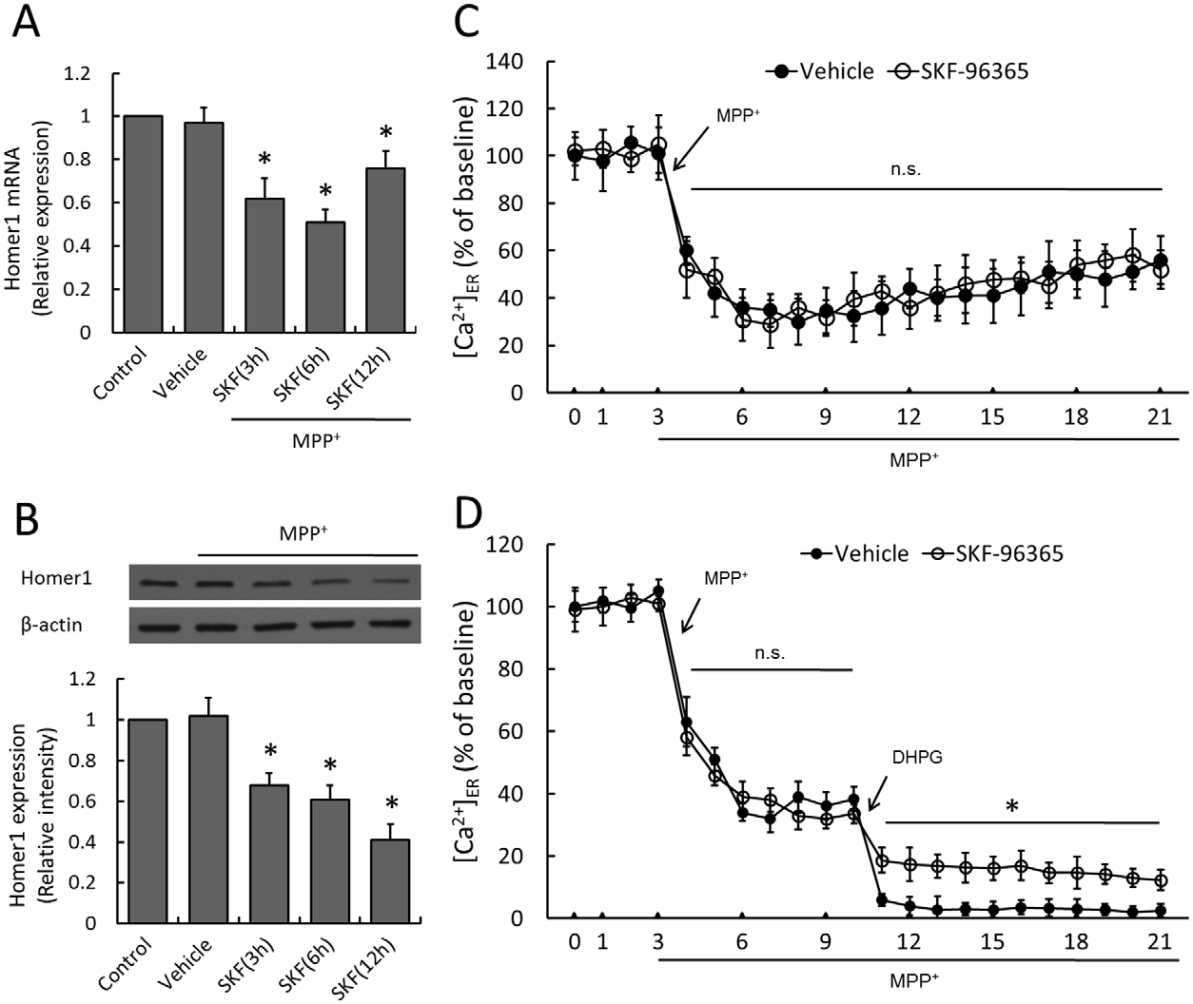
Effect of SKF-96365 on Homer1 expression and ER Ca^2+^ release. PC12 cells were pretreated with 50 µM SKF-96365 or saline water (Vehicle) 30 min before MPP^+^ insult, and the expression of Homer1 mRNA (A) or protein (B) was examined by real time RT-PCR or western blot analysis at 3 h, 6 h or 12 h, respectively. The Ca^2+^ concentration in ER ([Ca^2+^]_ER_) was measured up to 21 min (C). After potentiating group I mGluR activity with selective agonist DHPG at 10 min, [Ca^2+^]_ER_ was measured in MPP+-insulted PC12 cells with or without SKF-96365 (D). The data were represented as means ± SD from five experiments. *p<0.05 vs. Vehicle group. n.s., not statistically significant.

To investigate the potential role of Homer1 protein in PC12 cells stressed by MPP^+^ insult, real time RT-PCR and western blot analysis were used to measure the expression of Homer1 mRNA and protein after MPP^+^ administration with or without SKF-96365 pretreatment. As shown in Fig. 4A and 4B, MPP^+^ injury did not alter the expression of Homer1 mRNA or protein statistically as compared to Control group (*p*>0.05), while SKF-96365 pretreatment significantly decreased the expression of Homer1 mRNA and Homer1 protein from 3 h to 12 h in a time dependent manner. We then monitored changes in [Ca^2+^]_ER_, which was considered to be one of the most important modulating target of Homer1. MPP^+^ resulted in an immediate release of ER Ca^2+^ within 5 min that slowly increased for 6–21 min, whereas SKF-96365 had no effects on the dynamic changes of [Ca^2+^]_ER_ (Fig. 4C). We also used DHPG to potentiating group I mGluR activity at 10 min, and we found that DHPG further decreased [Ca^2+^]_ER_ from 11 min to 21 min, which was partly reversed by SKF-96365 (Fig. 4D).

**Figure 5:**
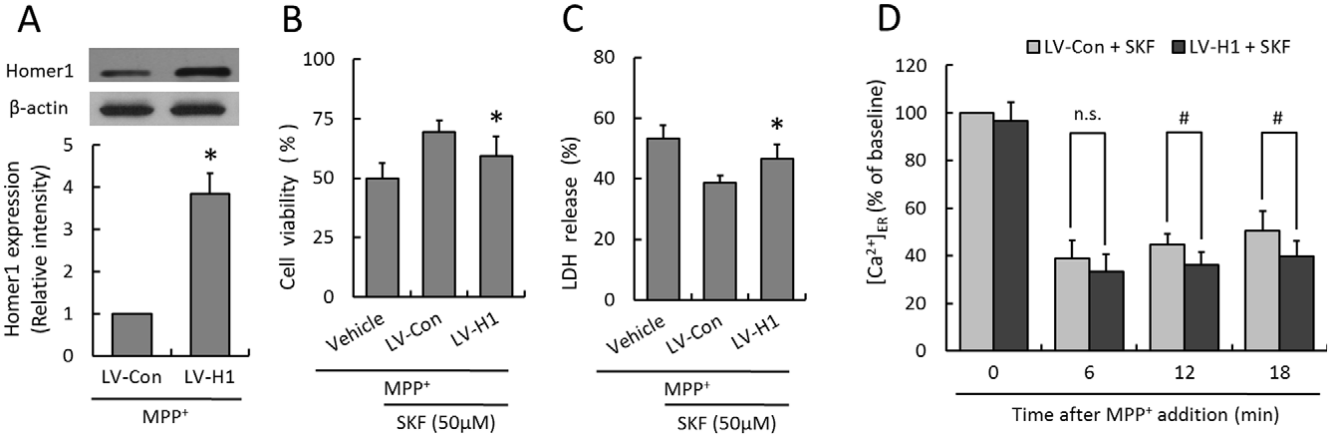
Effect of Homer1 overexpression on SKF-96365-induced neuroprotection. PC12 cells were transfected with lentivirus expressed Homer1 (LV-H1) or control lentivirus (LV-Con) for 72 h before MPP^+^ insult, and the expression of Homer1 was examined by western blot analysis (A). The cell viability (B) and LDH release (C) were assayed 24 h later, and the Ca^2+^ concentration in ER ([Ca^2+^]_ER_) was measured at 6, 12 and 18 min after MPP^+^ insult (D), respectively. The data were represented as means ± SD from five experiments (A, B and C) or eight experiments (D). *p<0.05 vs. control lentivirus (LV-Con), ^#^ *p*<0.05. and n.s., not statistically significant.

To further shed light on the relationship between protective effects of SKF-96365 and its suppressive activity on Homer1 expression, PC12 cells were transfected with lentivirus expressed Homer1 (LV-H1) and control lentivirus (LV-Con). Immunoblot analysis indicated that exogenous Homer1 was expressed in PC12 cells by transfection of LV-H1, and the total level of Homer1 was upregulated to about 4-fold of that in Vehicle cells (Fig. 5A). As shown in Fig. 5B and 5C, the protective effects of SKF-96365 on MPP^+^ induced cytotoxicity, as demonstrated by increased cell viability and decreased LDH release, were partly reversed after transfection with LV-H1. Moreover, the level of [Ca^2+^]_ER_ was markedly lowered by LV-H1 transfection as compared to LV-Con transfected PC12 cells at 12 min (44.6±4.6% vs 36.1±5.4%) and 21 min (50.3±8.3% vs 39.8±6.3%), indicating an amplified ER Ca^2+^ release and delayed calcium recovery in Homer1 upregulated PC12 cells following MPP^+^ administration (Fig. 5D).

## Discussion

PD affects about 2% of the population over the age of 60, and the incidence is expected to rise dramatically in the next 25 years with the extension of life expectancy by improved health care [19], [20]. Due to the difficulty to find a suitable and accessible model studies of PD in humans, many in vitro experimental models in cell lines are often used to investigate idiopathic PD with respect to molecular and neurochemical parameters [21], [22]. The PC12 cells have been shown to synthesize and excrete DA and to differentiate to neuron-like cells, and may serve as an in vitro model for studying neuronal damage in PD [23], [24]. In the present study, PC12 cells were treated with MPP^+^, a positive charged molecule with toxicity on oxidative phosphorylation in mitochondria, to mimic the neuronal injury in PD. It has been reported that MPP^+^ is toxic to DA neurons of mesencephalic cultures at concentrations as low as 0.5 to 2.0 µM, but shows effects in immortalized cell lines only at concentrations exceeding 100 µM [25], [26]. We found that MPP^+^ insult (at the concentration of 300 µM) increased the LDH release, decreased the cell viability, and caused karyopycnosis and karyorrhexis, as well as intracellular calcium overload in PC12 cells, indicating the acquisition of all desired morphological and biochemical characteristics of an in vitro cellular model to study PD.

The molecular mechanisms underlying the cellular death found in the nigrostriatal pathway during the progression of PD are not completely understood. Treatment of dopamine precursor levodopa, the most effective symptomatic treatment for PD is limited by unfavorable pharmacokinetic properties, and does not prevent the natural progression of PD and neurons continue to degenerate [27], [28]. All these facts indicate that neuroprotection of the native dopaminergic system by pharmacological agents may be a more robust strategy. In this study, we showed evidence that pretreatment with SKF-96365, a non-specific inhibitor of SOCE, exhibited protective activity against MPP^+^ injury in PC12 cells. Many in vitro studies have shown that both apoptosis and necrosis are involved in the pathway of neuronal death induced by MPP^+^ toxicity, which was also confirmed in our study. The results of Hoechst 33342 staining and flow cytometry showed that SKF-96365 significantly inhibited apoptotic cell death in PC12 cells after MPP^+^ administration. Interestingly, in spite of a distinct protective effect of SKF-96365 on cellular membrane damage as demonstrated by PI staining, the number of AV^−^/PI^+^ cells in SKF-96365 treated group was not significantly different from that in Vehicle group, indicating that SKF-96365 did not exert effects on necrotic cell death induced by MPP^+^ insult in PC12 cells. Further investigation seems to be necessary to confirm the findings and to elucidate whether it is an anti-apoptotic and necrosis-independent protective mechanism.

SKF-96365 was originally described as an inhibitor of voltage-gated and receptor-mediated Ca^2+^ entry over Ca^2+^ release from internal stores in several non-excitable cells [29]. Moreover, SKF-96365 is believed to be a non-selective blocker of TRPC3/6/7 channels, leading to its wide use as a tool-drug to define the functional roles of TRPC channels in various cell and tissue types [30], [31]. SKF-96365 is often used in the concentration range of 1–100 µM with IC_50_ values in range of 5–30 µM as previous reported [32]–[34]. We used the concentration of 1 µM, 10 µM and 50 µM in this experiment, and the results showed that 10 µM and 50 µM SKF-96365, but not 1 µM, were able to exert protective effects against MPP^+^-induced damage in PC12 cells. Due to its non-selective activity, SKF-96365 has been demonstrated to have effects on multiple other Ca^2+^ channels. For example, a previous study showed that SKF-96365 not only blocked high-voltage-activated (HVA) Ca^2+^ channels at typically utilized test concentrations, but also potently inhibited low-voltage -activated (LVA) T-type Ca^2+^ channels in HEK293 cells [35]. Therefore, the exact effect of SKF-96365 on intracellular calcium homeostasis might dependent on cell types and experimental models used. In our studies, SKF-96365 at the concentration of 50 µM apparently reduced the intracellular calcium overload induced by MPP^+^ insult, indicating a buffering effect on calcium overload in our in vitro PD model.

Homer proteins are originally identified as part of a complex scaffolding of proteins that comprise the postsynaptic density (PSD), and they are thought to be able to link cell surface receptors to multiple receptor proteins that regulate intracellular calcium homeostasis, such as IP_3_R and ryanodine receptors (RyR) [14], [36], [37]. Based on the presence of a conserved proline-rich sequence LP(P/X)PFN, which is similar to the consensus Homer biding site, in TRPC channels, the contribution of Homer to TRPC channel function has been comprehensively investigated. Homer1 gates the activity of TRPC1 channels, controls the rates of their translocation and retrieval from the plasma membrane, and mediates the conformational coupling of a TRPC1-Homer1-IP_3_R complex [16]. However, the exact role of SOCE channels activity on the modulation of Homer1 expression and function is poorly understood. In this study, we found that blocking SOCE channels by non-selective antagonist SKF-96365 significantly decreased the expression of Homer1 mRNA and protein in an in vitro model of PD. A previous study has shown that NMDA receptor stimulation and brain-derived neurotrophic factor (BDNF) upregulated Homer gene expression via calcium dependent activities of mitogen-activated protein kinases (MAPKs) cascade [38], and we also observed a similar pattern of Homer gene induction in vascular endothelial growth factor (VEGF) treated neurons [39]. Our results of this study indicate that SOCE channels mediated Ca^2+^ influx might also be at least in part involved in Homer1 modulation.

Intracellular Ca^2+^ increase under disease conditions usually results from two major mechanisms: one is the Ca^2+^ influx from extracellular space through Ca^2+^ channels across the plasma membrane such as TRPC channels, and the other is intracellular Ca^2+^ release from ER calcium stores [40]–[42]. Homer1 is required for activation of group I mGluR-IP_3_R-ER axis and followed Ca^2+^ release from ER to intracellular space, but it is interesting that in this study SKF-96365 reduced Homer1 expression with no effects on ER calcium concentration. So we potentiated group I mGluR activity with selective agonist DHPG at 10 min, and measured ER calcium concentration to further testing our hypothesis. We found that DHPG further decreased [Ca^2+^]_ER_ from 11 min to 21 min while DHPG partly reversed this effect, suggesting that SKF-96365 induced suppression of Homer1 expression inhibited group I mGluR-IP_3_R-ER Ca^2+^ axis mediated ER calcium release. This can be explained by the fact that SKF-96365 at the concentrations required to block store-operated calcium (SOC) entry (or referred to as capacitative calcium entry) may activate an intracellular calcium release [43], [44], which revised the preservation of ER calcium concentration mediated by SKF-96365 induced Homer1-group I mGluR-IP_3_R-ER Ca^2+^ axis inhibition. Moreover, upregulating the expression of Homer1 protein using recombinant lentivirus partly reversed protective effects of SKF-96365 against MPP^+^ induced cellular injury, and further amplified the Ca^2+^ release from ER. All these data confirmed that SKF-96365 not only decreased the expression of Homer1 protein, but also suppressed Homer1 mediated ER calcium release function through group I mGluR-IP_3_R-ER Ca^2+^ axis, which was reversed by side effect of SKF-96365 on intracellular calcium release.

In summary, our present study showed that the non-selective antagonist SKF-96365 protects PC12 cells against MPP^+^ induced cytotoxicity. Moreover, these protective effects were partly dependent on attenuating intracellular calcium overload, as well as inhibition of Homer1 expression and followed ER calcium release. These findings imply that blocking SOCE channels mediated intracellular calcium overload may have therapeutic potential for PD, which needs further determined by animal experiments.
